# Secondary-Structure-Informed RNA Inverse Design via Relational Graph Neural Networks

**DOI:** 10.3390/ncrna11020018

**Published:** 2025-02-26

**Authors:** Amirhossein Manzourolajdad, Mohammad Mohebbi

**Affiliations:** 1 Department of Computer Science, State University of New York Polytechnic Institute, 100 Seymour Rd., Utica, NY 13502, USA; 2 Department of Computer Science and Information Science, University of North Georgia, Dahlonega, GA 30597, USA; mmohebbi@ung.edu

**Keywords:** RNA inverse design, geometric deep learning, riboswitches, graph neural networks

## Abstract

RNA inverse design is an essential part of many RNA therapeutic strategies. To date, there have been great advances in computationally driven RNA design. The current machine learning approaches can predict the sequence of an RNA given its 3D structure with acceptable accuracy and at tremendous speed. The design and engineering of RNA regulators such as riboswitches, however, is often more difficult, partly due to their inherent conformational switching abilities. Although recent state-of-the-art models do incorporate information about the multiple structures that a sequence can fold into, there is great room for improvement in modeling structural switching. In this work, a relational geometric graph neural network is proposed that explicitly incorporates alternative structures to predict an RNA sequence. Converting the RNA structure into a geometric graph, the proposed model uses edge types to distinguish between the primary structure, secondary structure, and spatial positioning of the nucleotides in representing structures. The results show higher native sequence recovery rates over those of gRNAde across different test sets (eg. 72% vs. 66%) and a benchmark from the literature (60% vs. 57%). Secondary-structure edge types had a more significant impact on the sequence recovery than the spatial edge types as defined in this work. Overall, these results suggest the need for more complex and case-specific characterization of RNA for successful inverse design.

## 1. Introduction

RNA molecule design is essential for gene therapy, drug development, bio-sensing, synthetic biology, and, more recently, mRNA vaccines [1,2,3]. However, in deep learning explorations, protein sequence–structure analyses have enjoyed more advances than those for RNAs. These advances are in part due to the significant amount of available data on protein structures [4,5,6,7,8]. Apart from the paucity of data, other major factors posing challenges in RNA inverse design are RNA’s inherent flexibility and plasticity, in some cases its ability to form distinct conformations under different environmental factors, and non-unique structure–sequence mapping [9].

The structure-based design of RNAs capable of folding into alternative structures, known as RNA switches or riboswitches [10], is of great interest in biotechnology and therapeutics [11]. Probably representatives of an ancient regulatory mechanism [12,13] dating even long before proteins [14,15,16,17], riboswitches are usually located in the 5′ untranslated region (UTR) of an mRNA. They generally consist of a ligand-binding aptamer that, upon binding to the ligand or metabolite of interest, can allosterically alter the structure of the rest of the riboswitch, which also includes an expression platform, in turn regulating the transcription or translation of downstream genes in bacteria [10,18,19,20]. Currently categorized into 55 distinct classes, riboswitches enjoy great structural and functional diversity [21,22,23], as well as there being a variety of ligands that bind to them, helping cells regulate a great variety of chemicals essential to all forms of life [19,24]. It is speculated that additional classes of riboswitches exist not only in bacteria but also in eukaryotes [17,19]. Riboswitches are used in many therapeutic and biotechnological applications, including riboswitch-targeting antibiotics [25,26,27,28], designer gene control [29,30,31], molecular fuses [32,33], and research tools for exploring fundamental biological processes [34].

RNA inverse design has traditionally been based on RNA’s 2D or secondary structure [35]. More recent approaches such as libLEARNA [36] incorporate sequence and secondary-structure constraints to perform partial sequence design with an outstanding performance when applied on riboswitches. One of the more popular 2D inverse folding tools is the RNAinverse tool from the software package ViennaRNA, Version 2.6.4 [37], which predicts the complete sequence.

The 3D structure of the RNA molecule can also be used as a transitional step to increase the credibility of RNA sequence design [38]. The computational design of RNA directly from its 3D structure can be performed using the state-of-the-art physically based molecular modeling software Rosetta (https://rosettacommons.org/) [39]. Several deep learning approaches to 3D RNA inverse design have also been explored in the past few years based on graph neural network (GNN) architectures. Some of these methods include the representation-based learning approach RDesign [40] and the diffusion-model approach RiboDiffusion [41] that uses a Transformer-based sequence module. Another GNN-based model, gRNAde [42], which incorporates concepts from autoregressive GVP-GNN [43] and the protein sequence design tool ProteinMPNN [6], has been shown to achieve a higher native sequence recovery compared to that of Rosetta and RDesign on a set of benchmark sequences that include riboswitches and ribozymes [44]. The multi-state architecture of gRNAde software (https://github.com/chaitjo/geometric-rna-design) uses multiple structures from a single sequence in its training and design. The architectural design, however, is more geared towards aggregating multiple types of structural information rather than combining them, which could lead to a loss of information in certain cases. An approach that is based on combining information from multiple structural states, however, is yet to be fully explored. In this work, based on gRNAde, a secondary-structure-informed relational graph neural network (GNN) is proposed for RNA inverse design with an architecture that emphasizes learning from RNAs that specifically have distinct alternative structures, i.e., riboswitches. The model uses geometric vector perceptron GNN [43] layers and has a similar architecture to that of gRNAde [42,45].

## 2. Results

The proposed RNA inverse design is mainly based on defining different edge types. The Materials and Methods Section offers an extensive description of node and edge embeddings, encoder and decoder structures, as well as the overall model architecture. Unlike the primary- and secondary-structure edge types, however, the spatial edge types are not as deterministic and vary based on the choice of modeling. Here, the intention behind the spatial edges was to capture some of the RNA molecules’ three-dimensional patterns that were evolved beyond the secondary structural constraints. Here, a simple model is used based on the relative orientation of unpaired nucleotides. A general analysis of all of the spatial edge types is provided in Appendix A.2. Given the unevenness of the relative orientation distribution of the unpaired nucleotides (Figure A2a), it was speculated that introducing spatial edge types as defined above may contain certain hidden information about the RNA nucleotide positioning in 3D space.

**Figure 1:**
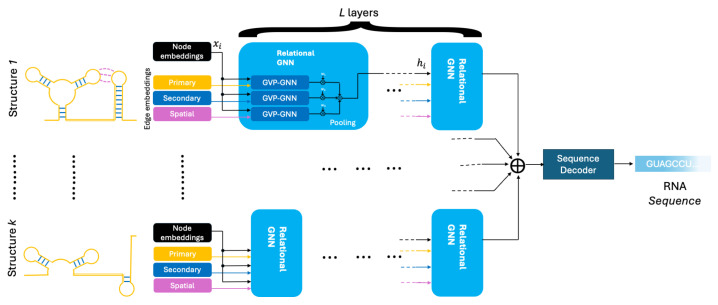
Secondary-structure-informed relational graph neural network diagram for the NAR-informed model. Node and edge embeddings of each structure *k* are fed into *L* cascaded layers of relational GNNs. Each layer is composed of three GVP-GNNs acting on their own primary-structure, secondary-structure, and spatial edge types, shown in orange, blue, and magenta, respectively. Messages are pooled to produce a single message used to update the node embedding before feeding it into the next layer. The final updated node embeddings corresponding to individual structures are then pooled to produce the final nucleotide probabilities using a linear model in the decoder.

The secondary-structure-informed relational GNN architecture used is shown in Figure 1. The model consists of *L* relational message-passing GNN layers. Each layer pools the messages from three distinct GVP-GNN components, with each corresponding to an edge type. Note that a single GVP-GNN component can be used instead of three, where ${\vec{e}}_{i,j}$ would be defined as the concatenation of ${{\vec{e}}^{p}}_{i,j}$, ${{\vec{e}}^{s}}_{i,j}$, and ${{\vec{e}}^{t}}_{i,j}$. In fact, Equations (2) and (3) are mathematically equivalent to Equation (1) from [42]. However, since the edges are mutually exclusive, merging of the edge types would result in a sparse adjacency matrix. In addition, each $e_{ij}$ vector would have been three times the original edge vector, padded by zeros in the corresponding locations. Although a single GVP-GNN would have been equivalent to the three original GVP-GNNs, deploying three GVP-GNNs had the advantage of requiring a smaller input size for the edges.

Given the multiple structures in a particular sequence, each individual sequence–structure pair is fed into the encoder separately, resulting in a *k* set of final updated node embeddings (the output of layer *L*). The node embeddings, with each resulting from a distinct structure, are then pooled before being fed into the decoder, which produces the final probabilities p(i) for each nucleotide *i*:

(1) $$p\left(i\right)=\left(1/K\right)\sum_{k\in K}p\left(i|structure_{k}\right)$$

Apart from the above re-structuring, the GVP-GNN components and linear and final decoder layers are adopted from gRNAde according to the software [42,45]. Two different decoders were introduced in [42] by the names of one-shot and autoregressive, which are referred to here as NAR-original and AR-original, respectively. The implementations for both models were altered to implement the above secondary-structure-informed relational GNN versions, denoted as NAR-informed and AR-informed, respectively. For each altered implementation, two different versions, *-informed-2D (The suffix 2D in *-informed-2D models should not be confused for two-dimensional. The model is still based on three-dimensional information about the canonical and non-canonical base pairings.) and *-informed-3D, were considered. In the case of AR-informed-*, pooling of the updated node embeddings was performed after autoregressive node prediction of the individual structure. Hence, Equation (1) holds for all implementations.

Table 1 contains a performance comparison between the original gRNAde and the secondary-structure-informed relational graph neural network under different data split strategies and for different parameters, such as the number of encoder layers and the inclusion/exclusion of spatial edge types.

The original autoregressive model AR-original, trained on the seqid data split and implemented as part of gRNAde, had a lower perplexity as well as a higher sc score across all models. The higher sc score is consistent across both sequence-based, seqid, and secondary-structure-based, structsim, data splits. Indeed, all of the AR-* models had a lower perplexity than that of their NAR-* counterparts. These relatively lower perplexity values are likely due to the fact that AR-* relies on part predictions (from $5^{\prime }$ to $3^{\prime }$) and predicts base pairing and base stacking interactions, essential to RNA’s structure, better, as opposed to NAR-*, which independently predicts the probabilities for the base pairs.

The NAR-* models generally had higher sequence recovery measures compared to those of their AR-* counterparts. These high recovery measures are also consistent with [45] and independent of our modeling architecture. We speculate the reason for this to be the autoregressive decoding algorithm. Due to the conditioned-prediction nature of AR-*, it learns to use different Watson–Crick base pairs, namely G-C and A-U, interchangeably, making the output probabilities more variable in the nucleotide positions corresponding to such base pairs than those of NAR-*. The sequence recovery measure is computed over multiple sequence samples, as opposed to the maximum-likelihood prediction. Hence, the base pair variability within the generated sequences leads to more mismatches with the ground truth. The relatively lower recovery of AR-* may not necessarily be a disadvantage in sequence design. In any case, as we can see in Table 1, all of the AR-* models have a higher prediction accuracy, i.e., maximum-likelihood prediction, than that of their NAR-* counterparts, with the seqid-trained four-layered AR-informed-3D model having the highest accuracy of 71.89 ± 0.3 overall.

Comparing the models based on the accuracy and recovery metrics, however, shows a higher performance for the *-informed-* models compared to that of their *-original counterparts. This higher performance is true regardless of the choice of data split strategy. In fact, the *-informed-* models only have two layers, while the original model is set to four layers. Finally, increasing the layers from two to four further improves the performance of the AR-informed-3D model to recovery = 58.37.

Comparing the performance of the *-informed-2D models to that of their *-informed-3D counterparts shows that the spatial edge types did not result in any major improvement.

The inverse design performance of the seqid-trained AR-informed-3D model was also evaluated on the 14 RNA structures of interest identified by [44] (their Supplementary Table S2) and compared to that of previous methods (see Table 2). The recovery values are based on the average accuracy of 16 predicted sequences in each case. The reason for choosing 16 samples was to have a consistent comparison with [45]. A total of six different inverse design strategies were applied to the 14 sequences. The six methods consisted of two 2D or secondary-structure-based approaches and four 3D or tertiary-structure-based approaches. The 2D inverse design approaches were the use of the RNAinverse tool from the ViennaRNA software package and the ant colony optimization-based tool antaRNA [46,47]. For 2D inverse design, the secondary structures were first determined from the PDB files using the DSSR program and then applied to the program. The 3D inverse design tools were RNADesign, Rosetta, gRNAde, and our proposed secondary-structure-informed approach, in particular seqid-trained AR-informed-3D, denoted in Table 2 as Ar-3D. The native sequence recovery for AR-3D had an overall value of 0.6044, which was slightly higher than that of gRNAde (0.5682) and also higher than that of the other methods, including Rosetta (0.45) and RDesign (0.4296). The recovery values for RNAinverse, RDesign, Rosetta, and gRNAde were taken from [45].

### The TPP Riboswitch, a Case Study for RNA Inverse Design

The choice of template structure. In order to showcase an example of RNA inverse design, we selected the thiamine pyrophosphate (TPP)-sensing riboswitch as a template. The ligand-bound structure of the *Arabidopsis thaliana* TPP riboswitch was resolved at a resolution of 2.9 Å using X-ray crystallography (PDB ID: 2CKY:A; length: 77 nt) [48].

Generating the sequence predictions. Four candidate predictions were selected for the analysis. The first two sequences were predicted using the seqid-trained AR-informed-3D model. The default output of the program was set to generate 16 predictions, which had an average recovery of 0.60 ± 0.05. From the predictions, we chose the sequence with the highest recovery, AR-3D-1, and the sequence with the highest sc score. The second two sequences were generated using solo-trained AR-informed-3D. The solo-trained model, specifically trained for this case study, only uses solo RNAs and DNA-RNA hybrids in its training, which is a much smaller dataset than the complete dataset (see the section on the data collection). The goal was to assess the performance of models trained only on solo RNAs (and RNA-DNA hybrids) versus that of those trained on all structural data, including RNA–protein complexes. The average recovery for the solo-trained predictions was 0.68 ± 0.04. The selected predictions were named AR-solo-1 and AR-solo-2, which corresponded to the highest recovery and the highest sc score predictions, respectively.

The choice of 3D structure prediction tool. Due to the absence of experimental data on the predicted sequences, the AlphaFold software (https://alphafold.ebi.ac.uk/) [49] was used to predict the 3D structures of the sequences. The AlphaFold web server provides four different predictions. We consistently selected the first prediction, namely model-0, for the analysis.

The choice of 3D structural similarity metric. The 3D structural similarity was measured by applying the Rclick algorithm [50] to the coordinates of the C4’ atoms of the structures. Rclick similarity is based on graph clique matching and 3D least squares fitting and has the advantage of detecting the local structural similarities independently of the topology. The program outputs both the RMSD and the percentage of the overlap between two given structures. The percentage of the overlap is more appropriate here since we are interested in the partial similarity between structures. Indeed, in many RNA inverse design applications, it is sufficient to obtain the similarity to the functional components of the template structure rather than a high similarity to its overall structure.

Two additional comparisons were added as benchmarks. We measured the structural overlap between 2CKY:A and the AlphaFold-predicted structure from its sequence. We also included the structural overlap measures between 2CKY:A and 2GDI:X [51], a homolog from an evolutionary distant organism *Escherichia coli*. The latter comparison was also included in [50] as a successful case study of identifying RNA-binding pockets.

Table 3 shows the sequences and the corresponding metrics of their comparison with the true structure of 2CKY:A. The secondary structures of 2CKY:A and 2GDI:X were determined from their PDB files using x3dna-dssr. The location marked as “[[” forms pseudoknots with chain Y of 2GDI. The secondary structures of the AR-*-* sequences were predicted from the sequences using the Maximum Expected Accuracy on the EternaFold web server [52]. The recovery values represent the accuracy of the sequence predictions to 2CKY:A. In the case of 2GDI:X, the nucleotides “GCG”, shown in red, were eliminated to obtain a consistent length, and then the recovery was calculated. The sc score values represent the self-consistency scores of the predicted secondary structures with the true secondary structure of 2CKY:A. In the case of 2GDI:X, the segment “[[”, shown in red, was eliminated to obtain a consistent length, and then the similarity of the secondary structures to 2CKY:A was computed. The columns “RMSD” and (percent of) “Overlap” represent the 3D structural similarity between the other predictions and 2CKY:A as assessed using Rclick. The values shown in “(( ))” represent the similarity between 2CKY:A and its AlphaFold prediction. In the case of 2GDI:X, the values represent the similarity between the true structure of 2GDI:X and that of 2CKY:A.

**Figure 2:**
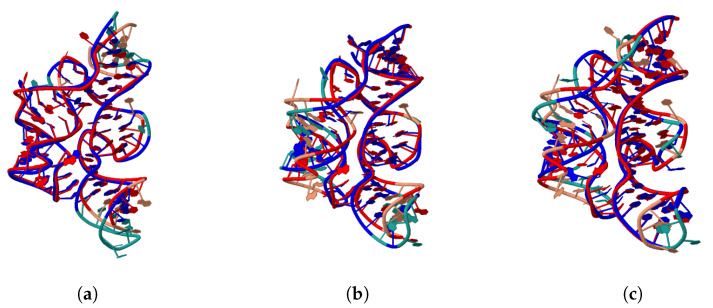
Structural alignment between 2CKY:A and the three structures (**a**) 2GDI:X, (**b**) AlphaFold-predicted 2CKY:A, and (**c**) AR-solo-1. The 2CKY:A structure is shown in salmon, and the other structures are shown in green. Aligned residues between any two pairs of structures are colored in red and blue. These figures were generated using the Rclick web server.

Despite having a fairly low sequence similarity of 0.65, 2GDI:X has the highest overlap with the 2CKY:A reference structure, at 83.12%. This high overlap highlights the degree of structural conservation in TPP riboswitches across distant species despite many mutations. The structural overlap between 2CKY:A and its own AlphaFold prediction is only 77.92%, illustrating the gap between true structures and the state-of-the-art prediction methods. Of the four predictions, AR-solo-1 had the highest percentage of overlap with 2CKY:A, at 76.62%, which was very close to the measure of the overlap of 2CKY:A with its own AlphaFold prediction. Figure 2a–c illustrate the structural overlaps between 2CKY:A and the three structures of 2GDI:X, the AlphaFold-predicted 2CKY:A, and AR-solo-1, respectively. The 2CKY:A structure is shown in salmon, and the other structures are shown in green. Aligned residues between any two pairs of structures are colored in red and blue. As we can see, the Y-shaped conformation of 2CKY:A is apparent in all three comparisons. The left helix of the Y-shape, however, has a better overlap with 2GDI:X (Figure 2a) compared to the other two predictions. The lower overlap in the left helix of Figure 2b seems to be an artifact of AlphaFold. The low overlap of the left helix is also visible in the case of AR-solo-1’s AlphaFold prediction, as shown in Figure 2b. Comparing Figure 2a–c, we can conclude that 76.62% is indeed a notably high overlap value and the lack of a perfect structural overlap is partly due to shortcomings in the 3D prediction. This case study shows that using the AR-solo model and selecting a prediction with the highest recovery can indeed lead to promising results for the RNA inverse design of TPP riboswitches. The performance of the AR-3D predictions (or AR-informed-3D), however, was lower than that of the AR-solo predictions, even though the AR-3D model used more training data in its optimization. The lower performance of AR-3D despite the higher amount of training data suggests that distinguishing the data types of 3D structures during model training can lead to improvements.

## 3. Materials and Methods

### 3.1. Data Collection

**Figure 3:**
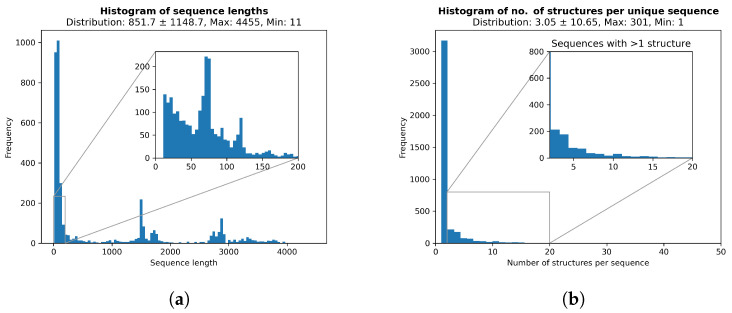
Data statistics. PDB files were downloaded from the RNAsolo web server [53] on 1 August 2024. (**a**) Histogram of sequence lengths. (**b**) Histogram of number of structures per unique sequence.

RNA structure files were downloaded from the RNASolo [53] repository on 1 August 2024. There were a total of 14,889 PDB files at a resolution ≤4 Å. The cleaned dataset contained a total of 12,091 structures, corresponding to 3963 unique sequences. The sequence types were as follows: 2612 protein–RNA complexes, 479 solo RNAs, 14 DNA-RNA hybrids, and 858 unknowns. The average sequence length was 851.74±1148.69. Only one structure was available for most sequences, and the number of structures per sequence followed an exponential distribution, with an average value of three structures per sequence. Figure 3a,b show the distributions of the sequence lengths and the number of structures per sequence, respectively. The similarity between structures from the same sequence was assessed by computing the average pairwise Root Mean Square Deviation (RMSD) of the structures belonging to that sequence. The average value was around 1.3 Å. Complete data statistics and histograms can be generated via notebooks/data_stats.ipynb. A machine learning ready set was then produced based on the downloaded PDB files. In cases where more than one structure was in the PDB file, the first one was used. Structures corresponding to the same RNA sequence were then categorized together, representing a multiple-structure, single-sequence set of data (see the section on the data availability for the location of the processed files).

### 3.2. Data Splits

Structures corresponding to protein–RNA complexes, solo RNAs, and DNA-RNA hybrids were all combined for training and testing throughout the analysis for consistency purposes. In the case study presented in the Results section, however, the AR-solo model only used solo RNAs and DNA-RNA hybrids for its training.

The RNA sequences were split into training, validation, and test sets. The sizes of the validation and test sets were 100 each. Two different strategies were used to split the data into training and test sets. The first one was denoted as seqid. In this procedure, the aim was to avoid exposing the model to too many different sequences during training. This was achieved by ensuring that the training set had less diverse sequences than the test set. The RNA sequences were clustered into groups based on sequence similarity using the CD-HIT command [54] (threshold = 90%) and, those with a lower intra-sequence RMSD among their structures were favored for the training set. In the second strategy, the aim was to avoid exposing the model to too many different structures during training. This was achieved by ensuring that the training set had less diverse structures than the test set. The RNA sequences were clustered into groups based on structural similarity using the command qTMclust [55] (threshold = 0.45%), and those with a lower intra-sequence RMSD among their structures were favored for the training set. Both data splitting procedures were performed according to [42].

### 3.3. Metrics

The performance parameters were perplexity, accuracy, recovery, and sc score. Perplexity reflects the ambiguity within the nucleotide probabilities. For instance, a model that predicts equi-probable nucleotides for each node is undesirable. A lower output perplexity is generally favorable (Note that a lower perplexity does not always reflect a superior model). It is possible to have a somewhat high perplexity/entropy for individual nucleotide probabilities but a low perplexity/entropy for their joint distributions. Accuracy is the ratio of correctly predicted nucleotides. Recovery is the ratio of correctly predicted nucleotides from a population of sequences that is sampled from the output probabilities. To ensure a consistent comparison of the performance with that of gRNAde, 16 predictions were used to calculate the sequence recovery. Similarly, sc score is the secondary-structure self-consistency score averaged over 16 predictions. The software Eternafold (https://eternafold.eternagame.org/) [52] was used to measure how well the predicted secondary structures of the sampled output sequences matched those of the ground-truth sequences.

### 3.4. Graph Construction of RNA Structures

**Figure 4:**
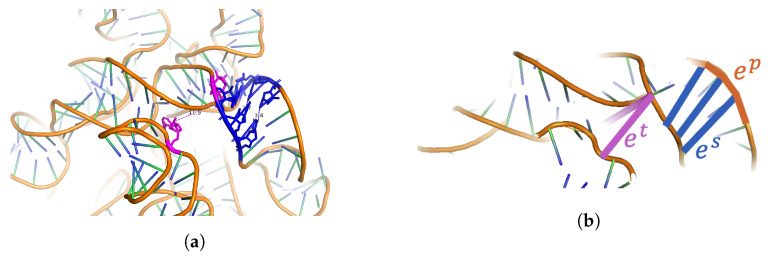
The three different edge types in an RNA structure graph. (**a**) The structure corresponds to part of the immature 30S ribosomal subunit from *Staphylococcus aureus*, 8BH7|1|a. Primary-structure edge types ($e^{p}$) are shown in orange, secondary-structure edge types ($e^{s}$) in blue, and spatial edge types ($e^{t}$) in magenta. Nucleotides corresponding to one of the spatial edges are colored in magenta. The PDB coordinates of the two nucleotides are C169 and A1457, and the distance between them is 11.9 Å. This figure was drawn using PyMOL [56]. (**b**) Schematic view of different edge types. Primary-structure edge types for one side of the helix are shown in orange. Secondary-structure edge types for three base pairs (six nucleotides) are shown in blue. Spatial edge types for two edges are shown in magenta.

The 3D coordinates of the atoms of an RNA structure, taken from its corresponding PDB file, were used to construct a geometric graph representation (nodes and edges) of the molecule. Three types of edges were defined and denoted as the primary-structure ${{\vec{e}}^{p}}_{i,j}\in E^{p}$, secondary-structure ${{\vec{e}}^{s}}_{i,j}\in E^{s}$, and spatial ${{\vec{e}}^{t}}_{i,j}\in E^{t}$ edge types between nodes *i* and *j*. An example of these edge types is illustrated in Figure 4, which corresponds to a section of the immature 30S ribosomal subunit of *Staphylococcus aureus*. Edge types are mutually exclusive: $E^{p}\cap E^{s}=Ø$, $E^{p}\cap E^{t}=Ø$, $E^{s}\cap E^{t}=Ø$.

#### 3.4.1. Nodes

The 3D coordinates for the P, C4’, N1 (pyrimidine), or N9 (purine) atoms in each nucleotide were used to define the corresponding node for that nucleotide [57]. The C4’ coordinates of nucleotide *i*, ${\vec{x}}_{i}\in R^{3}$, represented the 3D coordinates of the corresponding node *i*. The 3D coordinates of P, C4’, and N1/9, as well as the atoms of the adjacent nucleotides on the RNA backbone, were used for feature representation.

#### 3.4.2. Primary-Structure Edge Types ${{\vec{e}}^{p}}_{i,j}$

Pairs of consecutive nucleotides on the sequence, i.e., j=i+1, were assigned primary-structure edge types in both the forward ${\vec{x}}_{i+1}-{\vec{x}}_{i}$ and backward ${\vec{x}}_{i}-{\vec{x}}_{i-1}$ directions.

#### 3.4.3. Secondary-Structure Edge Types ${{\vec{e}}^{s}}_{i,j}$

Pairs of nucleotides in the secondary-structure RNA base pairings, (i,j)∈BP, were assigned secondary-structure edge types. For each base pairing (i,j), two edges ${\vec{x}}_{i}-{\vec{x}}_{j}$ and ${\vec{x}}_{j}-{\vec{x}}_{i}$ were defined. Here, the secondary-structure base pairings included both canonical and non-canonical pairings [58] and were determined by feeding the corresponding PDB file of an RNA to X3dna-dssr [59]. In addition to X3dna-dssr, other choices of software such as Fr3d (https://www.bgsu.edu/research/rna/software/fr3d.html) [60] and the corresponding implementation NA_pairwise_interactions.py can also be used to extract secondary-structure edge types. The Fr3d software annotates the base pair interactions in RNA 3D structures according to the Leontis–Westhof classification [58]. Using Fr3d led to similar results.

#### 3.4.4. Three-Dimensional [Spatial] Positioning Edge Types ${{\vec{e}}^{t}}_{i,j}$

Pairs of nucleotides that did not form secondary-structure base pairings but were in close proximity to each other were considered to have what is referred to here as spatial edge types. The procedure was as follows: Given the secondary-structure base pairs, the unpaired nucleotides were first identified, i.e., i,j∉BP, and then clustered using Density-Based Spatial Clustering of Applications with Noise (DBSCAN). The default parameters for DBSCAN were DBSCAN_eps = 20 Å and min_samples = 5. Subsequently, pairs of nucleotides that belonged to the same cluster with a distance of $d={∥{\vec{x}}_{i}-{\vec{x}}_{j}∥}_{2}<2\times$ DBSCAN_eps and that were also far apart in the RNA sequence were selected. A distance |j−i|≥500, or at least 500 nt, was considered far apart, i.e., a default primary_dist = 500. The number of original spatial edge types increased exponentially with the sequence length. The edges were randomly selected from the original sample such that the number of spatial edges was strictly less than or equal to 4×L, where *L* was the length of the corresponding RNA sequence. The reason for the above threshold was to obtain similar numbers of edges for all edge types.

The rationale behind the selection of the clustering algorithm: The original work gRNAde conducted by [42,45] applied the k-nearest neighbors (KNN) algorithm to the nucleotides in an RNA molecule using their three-dimensional positioning to determine the neighbors of each node. The gRNAde model then defined the edges between a nucleotide and its k-nearest neighbors, k = 32, by default. Although the edges in gRNAde include backbone and secondary-structure pairings, they are not limited to a specific length and do not always represent actual bonds. Following the same trend, the spatial edges in this work are not meant to capture any of the characteristics of the bonds between the nucleotides either, only their three-dimensional positioning. Indeed, the minimum length threshold for ${\vec{e}}^{t}$ is 2 Å. We chose, however, to use DBSCAN to cluster the nucleotides, as opposed to KNN in gRNAde. The reason for this was that we were more interested in capturing pockets that were surrounded by, but not filled by, nucleotides. While the ${\vec{e}}^{p}$ and ${\vec{e}}^{s}$ edge embeddings represent fixed-angle chemical bonds, the ${\vec{e}}^{t}$ embeddings have a more statistical interpretation due to the dynamic nature of the RNA molecule.

**Figure A1:**
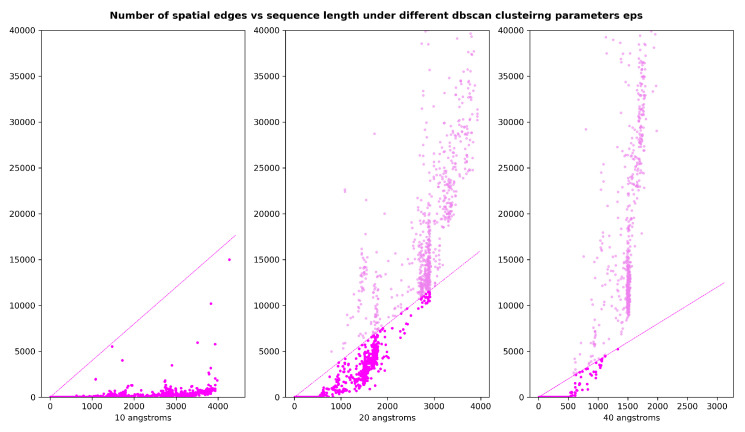
Number of spatial edges versus sequence length. Plots represent the number of edges under three different choices of DBSCAN eps clustering parameters: 10 Å, 20 Å, and 40 Å. The magenta dashed line shows the cut-off value for the number of spatial edges with respect to sequence length.

**Figure A2:**
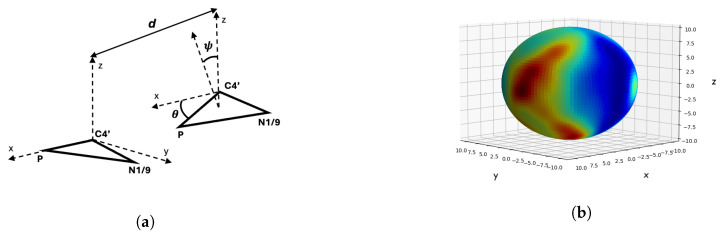
Relative orientations of distant nucleotide pairs with spatial edge types. (**a**) The angle ψ was defined as the angle between normal vectors of the two (C4’,P,N1/9) planes of the two nucleotides. The angle θ was defined as the angle between the two C4’-P vectors. (**b**) A density plot of the normalized relative orientation of around 43 thousand nucleotide pairs, taken from the RNA structures of the old dataset. All pairs had a Euclidean distance of 2≤d≤20 in Å. Red represents high-density and blue represents low-density orientations.

The rationale behind the selection of the clustering parameters: One of the major parameters in DBSCAN is DBSCAN_eps, which was set to 20 Å by default. The reason behind this value was partly algorithmic considerations. As we can see in Figure A1, setting the epsilon parameter to 10 Å or 40 Å leads to either too low (mostly zero) or too high (almost all possibilities) a number of edges, respectively. In order to capture the patterns in the spatial positioning of nucleotides on RNA molecules that were independent of canonical and non-canonical base pairings, we set a minimum distance threshold of primary_dist = 500 between the nucleotides on the RNA sequence for them to qualify as having a spatial edge between them. The parameters were not optimized further to avoid possible over-fitting.

**Figure 5:**
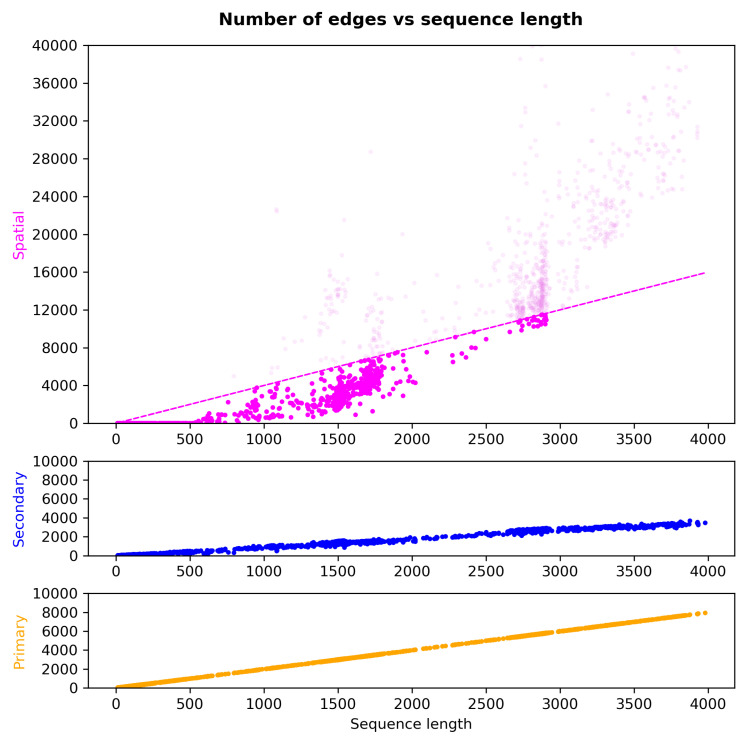
Number of primary-structure, secondary-structure, and spatial edge types with respect to sequence length. Light magenta represents the original counts of the spatial edges. The magenta dashed line shows the cut-off value for the number of spatial edges with respect to sequence length. RNAs with higher edge counts had their spatial edges down-sampled to strictly match the threshold.

The number of edges selected from each RNA molecule is shown in Figure 5. As we can see, the number of edges selected is linear with respect to the length of the sequence. The dashed magenta line shows the cut-off value for cases where the number of original edges was greater than the threshold. The edges of RNAs with higher counts were down-sampled to strictly match the threshold.

### 3.5. The Relational Graph Neural Network

#### 3.5.1. Input Embedding

Based originally on [43,61] and identical to the implementation presented in [45], the node features were derived from three distances, three inter-atom angles, and three dihedral angles. The numbers of scalar features associated with each node *i* were then the sin and cos values of the six angles concatenated by the three lengths, totaling 15 values. The vector features associated with each node *i* were four unit vectors corresponding to the forward and backward C4’ - P and forward and backward C4’ - N1/9 vectors. The scalar and vector features of node *i* are denoted as $\left(s_{i},{\vec{v}}_{i}\right)$. Edges of all types, ${\vec{e}}_{i,j}$, had identical features. The edge features consisted of both unit vectors and scalar values. Normalized vectors $\langle {\vec{e}}_{i,j}\rangle ={\vec{e}}_{i,j}/∥{\vec{e}}_{i,j}∥$ were used as unit vectors. The scalar features consisted of ${∥{\vec{x}}_{i}-{\vec{x}}_{j}∥}_{2}$ encoded by 16 RBFs. Let $x_{i}\in X$ represent both the scalar and vector features of node *i* and $e_{i,j}\in E$ represent both the scalar and vector features of edge (i,j). Hence, $E=\{E^{p},E^{s},E^{t}\}$. Let the matrix *T* represent the type of each edge in *E* in its corresponding indices. For a single sequence–structure scenario, each RNA structure is then represented as a graph G=(X,E,T). In cases of multiple (*K*) conformations, the graph corresponding to the structure *k* is denoted as $G^{\left(k\right)}=\left(X^{\left(k\right)},E^{\left(k\right)},T^{\left(k\right)}\right)$. All structures belonging to the same sequence, $\left\{G^{\left(1\right)},\cdots ,G^{\left(K\right)}\right\}$, are then passed into the GNN model in a single batch. Unlike [42], however, where the *K* graphs were merged into a single multi-graph with the total edges being the union of those of the individual structures, here, the edges of each structure *k* were applied to the model separately. Hence, although the number of nodes is identical amongst the *K* graphs, the number of edges is not. The rationale behind the above choice of input representation of multiple structures was to favor the expressiveness of an RNA molecule with drastically different conformational states, e.g., a riboswitch, over that of an RNA molecule with only one innate structure but with multiple experimental data on that structure.

#### 3.5.2. The Model Encoder

The overall architecture of the pipeline consisted of a linear model and *L* stacked layers of relational GNNs, followed by a decoder layer which produced the nucleotide probabilities for each sequence location *i*. The node and edge embeddings are first passed through the linear model. Encoded features are then passed to the first relational GNN layer. The relational GNN consists of three O(3)-equivariant GVP-GNN [43] components. Using the edge type indices, *T*, the encoded features of $G^{\left(k\right)}$ are first broken down into three sub-graphs, ${G^{p}}^{\left(k\right)}=\left(X^{\left(k\right)},{E^{p}}^{\left(k\right)}\right)$, ${G^{s}}^{\left(k\right)}=\left(X^{\left(k\right)},{E^{s}}^{\left(k\right)}\right)$, and ${G^{t}}^{\left(k\right)}=\left(X^{\left(k\right)},{E^{t}}^{\left(k\right)}\right)$, with each representing a graph of a specific edge type. Each of the sub-graph encodings is fed into a separate GVP-GNN (Equation (2)) and then pooled using learned weights (Equation (3)) to produce updated node features (Equation (4)):

(2) $$\begin{array}{cc} {m^{p}}_{i},{{\vec{m}}^{p}}_{i} & :=\sum_{j\in N_{i}^{p}}\mathrm{MSG}\left(\left(s_{i},{\vec{v}}_{i}\right),\left(s_{j},{\vec{v}}_{j}\right),{{\vec{e}}^{p}}_{i,j}\right)\\ {m^{s}}_{i},{{\vec{m}}^{s}}_{i} & :=\sum_{j\in N_{i}^{s}}\mathrm{MSG}\left(\left(s_{i},{\vec{v}}_{i}\right),\left(s_{j},{\vec{v}}_{j}\right),{{\vec{e}}^{s}}_{i,j}\right)\\ {m^{t}}_{i},{{\vec{m}}^{t}}_{i} & :=\sum_{j\in N_{i}^{t}}\mathrm{MSG}\left(\left(s_{i},{\vec{v}}_{i}\right),\left(s_{j},{\vec{v}}_{j}\right),{{\vec{e}}^{t}}_{i,j}\right) \end{array}$$

(3) $$m_{i},{\vec{m}}_{i}:=\sum_{e\in \left\{p,s,t\right\}}w^{e}\left({m^{e}}_{i},{{\vec{m}}^{e}}_{i}\right)$$

(4) $${s^{\prime }}_{i},{{\vec{v}}^{\prime }}_{i}:=\mathrm{UPD}\left(\left(s_{i},{\vec{v}}_{i}\right),\left(m_{i},{\vec{m}}_{i}\right)\right)$$

Messages with the *p*, *s*, and *t* indices reflect primary-structure, secondary-structure, and three-dimensional, or spatial, messages from neighboring nodes, respectively. The learned weights, $\left\{w^{p},w^{s},w^{t}\right\}$, reflect the significance of each edge type, i.e., the attention given to different edge types. The above message-passing and pooling mechanism applies to any relational GNN layer, and the layer indices are omitted for simplicity. For instance, the edge type weights of layer *l* can be denoted as $\left\{{w^{p}}^{\left(l\right)},{w^{s}}^{\left(l\right)},{w^{t}}^{\left(l\right)}\right\}$. In order to assess the impact of the secondary and tertiary edge types separately, two different versions of the model were used, one including the primary and secondary edge types only, denoted by the suffix -2D, and one including all of the edge types, denoted here by the suffix -3D.

The scalar and vector node features are updated at every layer (Equation (4)). For instance, the output of layer *l* is $\left({s^{\left(l\right)}}_{i},{{\vec{v}}^{\left(l\right)}}_{i}\right)$. The above message-passing scheme is applied to all *K* structural conformations in the input. At the end of the last encoder layer, the updated node embedding for the *k*-th structure is $\left({s^{\left(L\right)}}_{i,k},{{\vec{v}}^{\left(L\right)}}_{i,k}\right)$. The final node (or nucleotide) embedding given all *K* structures of the RNA sequence is then an average of the individual estimates:

(5) $$s_{i},{\vec{v}}_{i}:=\sum_{k\in K}\left({s^{\left(L\right)}}_{i,k},{\vec{v^{\left(L\right)}}}_{i,k}\right)$$

#### 3.5.3. The Model Decoder

The original model [42] presented two different decoder implementations: non-autoregressive, NAR-original, and autoregressive AR-original. The NAR-original decoder consisted of two GVP-GNN modules and decoded the node embedding into a dimension of four to reflect the four nucleotide probability estimates at each sequence position. The AR-original decoder was an autoregressive model [62] that updated each node embedding based on the nodes that preceded it in the sequence. The NAR decoder, on the other hand, follows a one-shot probability prediction architecture. Here, based on the above two decoders, two secondary-structure-informed decoders by the names of NAR-informed and AR-informed were deployed. The implementation of NAR-informed was identical to that of NAR-original. In the AR-informed decoder, node embeddings specific to the structure *k* are first independently updated, $\left(s_{i,k},{\vec{v}}_{i,k}\right)$. A final decode layer then pools the individual embeddings to generate the final nucleotide probabilities for each sequence position.

### 3.6. Architectural Differences from gRNAde

The edge embedding does not include 16 sinusoidal positional encodings. In addition, the edges consist of three different types: primary-structure edge types, secondary-structure edge types, and spatial edge types. The primary-structure edge types are defined along the sequence backbone. The secondary-structure edge types are determined by independent software [59,63] that detects canonical and non-canonical RNA base pairings. Spatial edge types have a specific definition here and are based on clustering the nucleotides based on a combination of their backbones and Euclidean distances. Each encoder layer consists of three distinct GVP-GNNs, with one for each edge type. Finally, the decoder takes updated node embeddings of the last layer of the encoder, with each resulting from a distinct structural scenario that the sequence can fold into, pooling them to produce the final updated node embedding. The proposed relational GNN is also O(3)-equivariant, and the message-passing components are implemented via PyTorch Geometric (https://pytorch.org) [64]. The Materials and Methods section describes the details about the input embedding and model architecture.

This paper first defines the model architecture in Materials and Methods. In the Results section, the performance of the relational GNN is then presented and compared to its corresponding gRNAde counterparts under different data splits and model variations. Finally, the Discussion section contains the conclusions drawn from the performance analyses and future directions.

## 4. Discussion and Conclusions

In this work, a geometric GNN-based model was presented to improve the computational design of RNA, especially that with multiple structural states, such as riboswitches. The proposed model, referred to as secondary-structure-informed RNA inverse design, is mostly taken from gRNAde, with some major differences. These differences were the use of different edge types to represent the RNA structural graphs and explicitly combining the node and edge embeddings of multiple structures after passing them the encoder, as opposed to aggregating them prior to the encoder. The details about these differences are explained in Materials and Methods. It was shown that the proposed model does indeed improve both the accuracy and native sequence recovery compared to those of gRNAde. Using the seqid training/test sets, the autoregressive AR-informed-2D model with only two layers obtained 71.12% sequence recovery, while its gRNAde counterpart with four layers produced 66.08% recovery. Similar improvements were seen under the structsim training/test sets (comparing 68.61% with 62.72%), as well as over different choices of decoders (see Table 1). These results demonstrate that the addition of secondary-structure base pairing information in RNA inverse design and combining the predictions via a relational graph architecture did indeed improve the performance since much of the model architecture between the structure-informed approach and gRNAde, as well as the default parameters, was kept intact. 

These improvements may also have been due to the fact that the identification of canonical and non-canonical base pairings, namely the 12 possible orientations [58], was carried out independently, relieving the GNN model of the burden of identifying such edges.

The performance of AR-informed-3D was also evaluated against a different benchmark and compared to that of other RNA design tools. The dataset presented in [44] contained 14 structures which included riboswitches and ribozymes. It is shown in Table 2 that the proposed model achieved a higher average native sequence recovery on this set over that of previous methods. The AR-informed model achieved a 60.44 native sequence recovery rate, higher than gRNAde’s (56.82), as well as that of Rosetta (45) and RDesign (42.96). These results highlight the merits of the proposed approach in the inverse design of novel structures.

The type of data fed into prediction models can have a major impact on the performance in RNA computational design. As we can see from the TPP riboswitch case study, a model trained only on solo RNAs (and RNA-DNA hybrids) outperformed the AR-informed-3D model trained on much more data, including RNA–protein complexes (comparing a sequence recovery of 0.68 to 0.60). In addition, as we can see in Table 3 and Figure 2, the AR-solo model prediction achieved a 76.26% 3D structural overlap with 2CKY:A, only slightly less than 2CKY:A’s overlap with its own AlphaFold-predicted structure (77.92%). The case study demonstrates that if the training data are carefully chosen, the proposed model is capable of successful RNA inverse design.

### Future Work

In order to identify RNA base pairings, we used X3dna-dssr [59]. Therefore, unlike gRNAde, which is a stand-alone software, our model relies on an external component to identify the secondary structural base pairing patterns. The performance measures presented here are only valid if the secondary structure of the desired RNA can be independently determined. gRNAde has the advantage of being able to handle the general case of only using the 3D structure to design sequences. In order to eliminate the need for external software, Python packages (https://www.python.org/) such as rnaglib [65] could be used to provide a graph representation of RNA 3D structures, with the edges representing base pairs and interactions.

The metrics recovery and sc score evaluate the primary- and secondary-structure similarity with the ground truth and are only generic assessments. Two RNAs with dissimilar sequence and secondary-structure predictions might still have a very similar tertiary structure. A gold-standard metric for effective RNA inverse design should be based on the similarity in key tertiary structural components. Reliable 3D structural prediction tools, such as the state-of-the-art [49], combined with effective similarity measures, such as [50], could be more effective in evaluating the design performance. To evaluate the successful design of RNAs with more than one structure, however, a more suitable metric may be needed.

The autoregressive AR-informed model led to the highest performance, supporting the fact that including autoregression is very effective in RNA inverse design. In this work, autoregression and pooling were carried out for each structure separately, only to combine the predictions at the last step, in Equation (1). One direction for improvement would be to use more effective autoregressive techniques. For instance, autoregression and pooling could be combined for each iteration *i*, i.e., determining p(i) from all *K* structures and only then repeating the process for p(i+1). Exploring novel, possibly *non-causal*, autoregressive strategies may be another avenue for improvement.

The incorporation of spatial edge types here did not lead to any major performance increase. The lack of a major improvement may partly have been due to the fact that there were simply fewer longer RNAs available in our dataset (see Figure 3) since spatial edges can only exist for sequences longer than 500 nt. It may also have been due to the fact that our spatial edge determination was too simplistic and could not capture any meaningful patterns in the RNA pockets or the spatial positioning of the nucleotides. Indeed, RNA 3D structural data such as base pair geometry and protein binding sites have been successfully used to predict the corresponding RNA’s function [65], which highlights the usefulness of extracting more complex structural patterns from RNAs. More effective modeling of RNA complexes, for example, labeling the ligand-binding configurations, may lead to identifying learnable and generalizable 3D patterns, potentially improving the performance for more complex RNA inverse design challenges.

In the case study presented here, it was shown that a model trained only on solo RNAs, although with much less data, could at times outperform a model that was trained on a larger amount of data that included different complexes. The lack of success of the AR-informed-3D model compared to that of the AR-solo model in the case study partly suggests the heterogeneous nature of RNA computational design and the need for customized models rather than a universal one. In addition, Table 2 showed a relatively acceptable performance for the AR-informed-3D model for the 14 sequences, which were all less than 200 nt. The performance was solely due to primary- and secondary-structure positioning information, as no spatial edges existed within the structures (minimum length: 500 nt). Hence, different model architectures and training strategies may be more effective when designing shorter RNAs versus longer RNAs.

The data on RNA structures that are currently available do not fully capture the different structural states of RNAs. Most experimental data are related the ligand-bound states of riboswitches, with apo (unbound) structures only occasionally being available. Multi-state geometric deep learning approaches such as gRNAde and the subsequent secondary-structure-informed alteration presented here may demonstrate heightened potential for inverse design once more data about the different structural states of an RNA molecule become available. Fortunately, recent advances in cryogenic electron microscopy (cryo-EM) are very promising for exploring RNA dynamics within three-dimensional space [66]. The incorporation of atomic-resolution data on biologically active RNAs, coupled with more new ways to interpret structural information on RNA, may lead to overcoming the current limitations in RNA inverse design.

## Figures and Tables

**Table 1:** Model performance comparison between the original gRNAde and the secondary-structure-informed relational graph neural network. Performance corresponds to 100 epochs. Standard deviations are reported across 3 seeds for all models. Abbreviations: Ppl. = Perplexity; Acc. = Accuracy; Rec. = Recovery; SC = sc score.

Model	Split	Layers	Ppl. (↓)	Acc. (↑)	Rec. (↑)	SC (↑)
AR-original	seqid	4	1.2501 ± 0.04	66.08 ± 0.30	50.84 ± 3.0	63.18 ± 3.01
AR-informed-2D	seqid	2	1.4875 ± 0.04	71.12 ± 0.60	54.51 ± 0.01	53.00 ± 0.04
AR-informed-3D	seqid	2	1.4604 ± 0.04	71.10 ± 0.60	54.08 ± 0.01	51.85 ± 0.04
AR-informed-3D	seqid	4	1.4023 ± 0.04	71.89 ± 0.30	56.83 ± 1.30	53.30 ± 0.31
AR-original	structsim	4	1.4843 ± 0.04	62.72 ± 0.30	44.85 ± 3.01	55.41 ± 3.0
AR-informed-2D	structsim	2	1.3422 ± 0.04	68.61 ± 0.60	50.31 ± 0.01	51.06 ± 0.04
AR-informed-3D	structsim	2	1.3471 ± 0.04	68.66 ± 0.60	49.40 ± 0.01	51.59 ± 0.04
AR-informed-3D	structsim	4	1.2984 ± 0.04	69.02 ± 0.30	50.23 ± 1.31	54.57 ± 0.30
NAR-original	seqid	4	1.5790 ± 0.10	53.95 ± 0.01	53.62 ± 2.01	38.68 ± 3.00
NAR-informed-2D	seqid	2	1.6062 ± 0.04	59.87 ± 0.60	61.06 ± 0.01	42.44 ± 0.04
NAR-informed-3D	seqid	2	1.4562 ± 0.04	60.17 ± 0.60	61.92 ± 0.01	30.66 ± 0.04
NAR-informed-3D	seqid	4	1.5483 ± 0.01	61.02 ± 0.01	61.40 ± 0.10	48.40 ± 0.01
NAR-original	structsim	4	1.9695 ± 0.10	47.18 ± 0.01	43.51 ± 2.00	39.36 ± 3.01
NAR-informed-2D	structsim	2	1.4444 ± 0.04	56.25 ± 0.60	55.07 ± 0.01	25.22 ± 0.04
NAR-informed-3D	structsim	2	1.4398 ± 0.04	54.97 ± 0.60	51.44 ± 0.01	26.37 ± 0.04
NAR-informed-3D	structsim	4	1.3413 ± 0.01	56.59 ± 0.01	53.00 ± 0.10	40.83 ± 0.01

**Table 2:** Performance comparison for the 14 structures of interest ([44], Supplementary Table S2). AR-informed refers to the seqid-trained AR-informed-3D model with four layers. The recovery values for AR-informed were computed over 16 sampled sequences for each structure. All the other recovery values are given according to [45]. Abbreviations: ViennaRNA = the RNAinverse function from ViennaRNA and AR-3D = seqid-trained AR-informed-3D.

PDB	Description	2D		3D			
		ViennaRNA	antaRNA	RDesign	Rosetta	gRNAde	AR-3D
1CSL	RRE high-affinity site	0.25	0.33	0.4455	0.44	0.5719	0.4263
1ET4	RNA aptamer	0.25	0.29	0.3929	0.44	0.6250	0.4379
1F27	RNA pseudoknot	0.30	0.23	0.3013	0.37	0.3437	0.3750
1L2X	RNA pseudoknot	0.24	0.18	0.3727	0.48	0.4721	0.5765
1LNT	internal loop of SRP	0.33	0.33	0.5556	0.53	0.5843	0.7131
1Q9A	sarcin/ricin dom.	0.27	0.63	0.4417	0.41	0.5044	0.8079
4FE5	guanine riboswitch	0.29	0.29	0.4112	0.36	0.5300	0.7687
1X9C	all-RNA hairpin ribozyme	0.26	0.27	0.3967	0.50	0.5000	0.3927
1XPE	HIV-1 B RNA	0.27	0.35	0.3834	0.40	0.7037	0.4266
2GCS	glmS ribozyme	0.25	0.28	0.4518	0.44	0.5078	0.6659
2GDI	TPP riboswitch	0.25	0.26	0.3523	0.48	0.6500	0.7680
2OEU	junctionless hairpin riboz.	0.23	0.27	0.5000	0.37	0.9519	0.7680
2R8S	tetrahymena ribozyme	0.27	0.33	0.5641	0.53	0.5689	0.6985
354D	loop E	0.28	0.38	0.4458	0.55	0.4410	0.8210
	Overall recovery:	0.27	0.32	0.4296	0.45	0.5682	0.6044

**Table 3:** Designed sequences and the corresponding metrics of their comparison with the template structure of 2CKY:A. Secondary structures of 2CKY:A and 2GDI:X were determined from their PDB files using x3dna-dssr. The secondary structures of the AR-*-* sequences were predicted from sequences using the Maximum Expected Accuracy on the EternaFold web server. The recovery values represent the accuracy of the sequence measures to 2CKY:A. The sc score values represent the self-consistency scores of the predicted secondary structures of the predicted sequences with the true secondary structure of 2CKY:A. The columns “RMSD” and “Overlap” represent the 3D structural similarity between the AlphaFold predictions and 2CKY:A as assessed using Rclick. Values shown in “(( ))” represent the similarity between 2CKY:A and its AlphaFold prediction. In the case of 2GDI:X, the values represent the similarity between the true structure of 2GDI:X and that of 2CKY:A.

Name	Sequence	Recovery	SC Score	RMSD	Overlap
2CKY:A	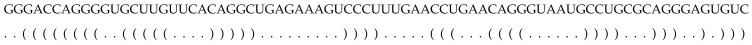			((2.22))	((77.92))
2GDI:X	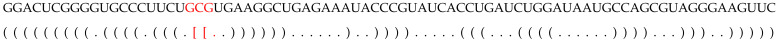	0.65	0.88	1.61	83.12
AR-3D-1	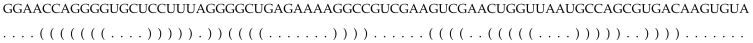	0.70	0.32	2.63	46.75
AR-3D-2	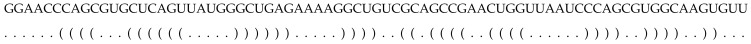	0.63	0.51	2.54	41.56
AR-solo-1	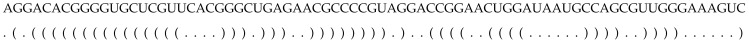	0.77	0.49	2.36	76.62
AR-solo-2	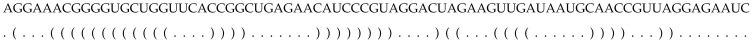	0.68	0.72	2.40	45.45

## Data Availability

The source code is available at https://github.com/amanzour/structure-informed-RNA-inverse-design.

## Abbreviations

The following abbreviations are used in this manuscript:

RNA	Ribonucleic acid
GNN	Graph neural network
GVP-GNN	Geometric vector perceptron graph neural network
3D	Three-dimensional
2D	Two-dimensional
UTR	Untranslated region
gRNAde	Geometric Deep Learning for 3D RNA inverse design
PDB	Protein Data Bank
Å	Ångström (unit of length)
O(3)-equivariant	Equivariant under the orthogonal group in three dimensions
PyMOL	Molecular visualization system
PyTorch Geometric	Library for graph neural networks
libLEARNA	Library for solving the partial RNA design problem
DNA	Deoxyribonucleic acid
RMSD	Root Mean Square Deviation
DBSCAN	Density-Based Spatial Clustering of Applications with Noise
RBF	Radial Basis Function
CD-HIT	Cluster Database at High Identity with Tolerance
qTMclust	Structure Clustering by Sequence-Independent Structure Alignment
X3dna-dssr	An integrated software tool for dissecting the spatial structure of RNA
Eternafold	RNA secondary-structure prediction software
sc score	Secondary-structure self-consistency score
NAR	Non-autoregressive
AR	Autoregressive
BP	Set of base pairs
nt	Nucleotide
TPP	Thiamine pyrophosphate
Ppl.	Perplexity
Acc.	Accuracy
Rec.	Recovery
SC	Secondary-structure compatibility score
DSSR	An integrated software tool for dissecting the spatial structure of RNA
X3DNA	A software package for the analysis and visualization of 3D nucleic acid structures
Fr3d	A software package for finding small RNA motifs in RNA 3D structures
AR-informed-2D	Autoregressive structure-informed model based on primary- and secondary-structure edge types
AR-informed-3D	Autoregressive structure-informed model based on primary-structure, secondary-structure, and spatial edge types
seqid	Sequence-based data split
structsim	Structure-based data split
Pred	Prediction
AlphaFold	Algorithm for protein and RNA 3D structure prediction
ProteinMPNN	Protein Message Passing Neural Network
Rclick	Tool for finding the overlap between two RNA 3D structures

## Appendix A

### Appendix A.1. The Impact of the DBSCAN Clustering Parameter (Eps) on the Number of Spatial Edges

Using all of the RNA structures available in the dataset, all nucleotide pairs with spatial edge types between them were identified under different choices of DBSCAN eps parameters. The number of spatial edges was computed under esp parameters of 10 Å, 20 Å, and 40 Å, shown from left to right in Figure A1, respectively. The magenta dashed line represents the cut-off value for the number of edges that are incorporated for the RNA.

### Appendix A.2. An Exploration into the Spatial Edge Type Characteristics

Using all of the RNA structures available in the dataset, all nucleotide pairs with spatial edge types between them were identified. The Euclidean distance and relative orientation between each of the corresponding node pairs were considered for a further analysis. The value *d* was a scalar representing the Euclidean distance between the C4’ coordinates of the two nucleotides. Filtering for a Euclidean distance of 2≤d≤20 in Å resulted in around 43 thousand such pairs. As illustrated in Figure A2a, the relative orientation was defined in terms of the spherical coordinates, with ψ being the angle from the positive z-axis and θ being the angle between the radial line and a polar axis. The angle ψ was defined as the angle between normal vectors of the two (C4’,P,N1/9) planes of the two nucleotides. The angle θ was defined as the angle between the two C4’-P vectors. A density plot of the normalized relative orientation of the nucleotide pairs is shown in Figure A2b. Red represents high-density and blue represents low-density orientations. Certain relative pairwise orientations were extremely rare in 3D space. In particular, relative orientations with an angle θ≈90° were not found, regardless of the values of ψ or *d*. On the other hand, certain orientations, such as ψ=70°, were more prevalent, representing an uneven distribution of relative orientations.

## References

[1] Doudna J.A., Charpentier E. (2014). The new frontier of genome engineering with CRISPR-Cas9. Science.

[2] Pardi N., Hogan M.J., Porter F.W., Weissman D. (2018). MRNA Vaccines—A New Era in Vaccinology. Nat. Rev. Drug Discov. Rev. Drug Discov..

[3] Metkar M., Pepin C.S., Moore M.J. (2024). Tailor made: The art of therapeutic mRNA design. Nat. Rev. Drug Discov..

[4] Bronstein M.M., Bruna J., Cohen T., Veličković P. (2021). Geometric Deep Learning: Grids, Groups, Graphs, Geodesics, and Gauges. arXiv.

[5] Jumper J., Evans R., Pritzel A., Green T., Figurnov M., Ronneberger O., Tunyasuvunakool K., Bates R., Žídek A., Potapenko A. (2021). Highly accurate protein structure prediction with AlphaFold. Nature.

[6] Dauparas J., Anishchenko I., Bennett N., Bai H., Ragotte R.J., Milles L.F., Wicky B.I.M., Courbet A., de Haas R.J., Bethel N. (2022). Robust deep learning-based protein sequence design using ProteinMPNN. Science.

[7] Watson J.L., Juergens D., Bennett N.R., Trippe B.L., Yim J., Eisenach H.E., Ahern W., Borst A.J., Ragotte R.J., Milles L.F. (2023). De novo design of protein structure and function with RFdiffusion. Nature.

[8] Duval A., Mathis S.V., Joshi C.K., Schmidt V., Miret S., Malliaros F.D., Cohen T., Liò P., Bengio Y., Bronstein M. (2024). A Hitchhiker’s Guide to Geometric GNNs for 3D Atomic Systems. arXiv.

[9] Vicens Q., Kieft J.S. (2022). Thoughts on how to think (and talk) about RNA structure. Proc. Natl. Acad. Sci. USA.

[10] Mandal M., Breaker R.R. (2004). Gene regulation by riboswitches. Nat. Rev. Mol. Cell Biol..

[11] Leppek K., Das R., Barna M. (2018). Functional 5’ UTR mRNA structures in eukaryotic translation regulation and how to find them. Nat. Rev. Mol. Cell Biol..

[12] White H.B.I. (1976). Coenzymes as fossils of an earlier metabolic state. J. Mol. Evol..

[13] Benner S.A., Ellington A.D., Tauer A. (1989). Modern metabolism as a palimpsest of the RNA world. Proc. Natl. Acad. Sci. USA.

[14] Nahvi A., Sudarsan N., Ebert M.S., Zou X., Brown K.L., Breaker R.R. (2002). Genetic control by a metabolite binding mRNA. Chem. Biol..

[15] Vitreschak A.G., Rodionov D.A., Mironov A.A., Gelfand M.S. (2004). Riboswitches: The oldest mechanism for the regulation of gene expression?. Trends Genet..

[16] Breaker R.R. (2009). Riboswitches: From ancient gene-control systems to modern drug targets. Future Microbiol..

[17] Breaker R.R. (2012). Riboswitches and the RNA world. Cold Spring Harb. Perspect. Biol..

[18] Sherwood A.V., Henkin T.M. (2016). Riboswitch-mediated gene regulation: Novel RNA architectures dictate gene expression responses. Annu. Rev. Microbiol..

[19] McCown P.J., Corbino K.A., Stav S., Sherlock M.E., Breaker R.R. (2017). Riboswitch diversity and distribution. RNA.

[20] Breaker R.R. (2018). Riboswitches and translation control. Cold Spring Harb. Perspect. Biol..

[21] Roth A., Breaker R.R. (2009). The structural and functional diversity of metabolite-binding riboswitches. Annu. Rev. Biochem..

[22] Serganov A., Nudler E. (2013). A Decade of Riboswitches. Cell.

[23] Peselis A., Serganov A. (2014). Themes and variations in riboswitch structure and function. Biochim. Biophys. Acta.

[24] Breaker R.R. (2022). The biochemical landscape of riboswitch ligands. Biochemistry.

[25] Blount K.F., Breaker R.R. (2006). Riboswitches as antibacterial drug targets. Nat. Biotechnol..

[26] Deigan K.E., Ferré-D’Amaré A.R. (2011). Riboswitches: Discovery of drugs that target bacterial gene-regulatory RNAs. Acc. Chem. Res..

[27] Mehdizadeh Aghdam E., Hejazi M.S., Barzegar A. (2016). Riboswitches: From living biosensors to novel targets of antibiotics. Gene.

[28] Panchal V., Brenk R. (2021). Riboswitches as drug targets for antibiotics. Antibiotics.

[29] Suess B., Weigand J.E. (2008). Engineered riboswitches: Overview, problems and trends. RNA Biol..

[30] Link K.H., Breaker R.R. (2009). Engineering ligand-responsive gene-control elements: Lessons learned from natural riboswitches. Gene Ther..

[31] Schmidt C.M., Smolke C.D. (2019). RNA switches for synthetic biology. Cold Spring Harb. Perspect. Biol..

[32] Wickiser J.K., Winkler W.C., Breaker R.R., Crothers D.M. (2005). The speed of RNA transcription and metabolite binding kinetics operate an FMN riboswitch. Mol. Cell.

[33] Ariza-Mateos A., Nuthanakanti A., Serganov A. (2021). Riboswitch mechanisms: New tricks for an old dog. Biochemistry.

[34] Kavita K., Breaker R.R. (2023). Discovering riboswitches: The past and the future. Trends Biochem. Sci..

[35] Churkin A., Retwitzer M.D., Reinharz V., Ponty Y., Waldispühl J., Barash D. (2018). Design of RNAs: Comparing programs for inverse RNA folding. Brief. Bioinform..

[36] Runge F., Franke J., Fertmann D., Backofen R., Hutter F. (2024). Partial RNA design. Bioinformatics.

[37] Lorenz R., Bernhart S.H., Höner zu Siederdissen C., Tafer H., Flamm C., Stadler P.F., Hofacker I.L. (2011). ViennaRNA Package 2.0. Algorithms Mol. Biol..

[38] Antczak M., Szachniuk M. (2025). Toward increasing the credibility of RNA design. Methods Mol. Biol..

[39] Leman J.K., Weitzner B.D., Lewis S.M., Adolf-Bryfogle J., Alam N., Alford R.F., Aprahamian M., Baker D., Barlow K.A., Barth P. (2020). Macromolecular modeling and design in Rosetta: Recent methods and frameworks. Nat. Methods.

[40] Tan C., Zhang Y., Gao Z., Hu B., Li S., Liu Z., Li S.Z. RDesign: Hierarchical Data-efficient Representation Learning for Tertiary Structure-based RNA Design. Proceedings of the The Twelfth International Conference on Learning Representations.

[41] Huang H., Lin Z., He D., Hong L., Li Y. (2024). RiboDiffusion: Tertiary structure-based RNA inverse folding with generative diffusion models. Bioinformatics.

[42] Joshi C.K., Jamasb A.R., Viñas R., Harris C., Mathis S., Liò P. Multi-State RNA Design with Geometric Multi-Graph Neural Networks. Proceedings of the ICML 2023 Workshop on Computation Biology.

[43] Jing B., Eismann S., Suriana P., Townshend R.J.L., Dror R.O. (2020). Learning from Protein Structure with Geometric Vector Perceptrons. arXiv.

[44] Das R., Karanicolas J., Baker D. (2010). Atomic accuracy in predicting and designing noncanonical RNA structure. Nat. Methods.

[45] Joshi C.K., Jamasb A.R., Viñas R., Harris C., Mathis S.V., Morehead A., Anand R., Liò P. (2024). gRNAde: Geometric Deep Learning for 3D RNA inverse design. bioRxiv.

[46] Kleinkauf R., Mann M., Backofen R. (2015). antaRNA: Ant colony-based RNA sequence design. Bioinformatics.

[47] Kleinkauf R., Houwaart T., Backofen R., Mann M. (2015). antaRNA–Multi-objective inverse folding of pseudoknot RNA using ant-colony optimization. BMC Bioinform..

[48] Thore S., Leibundgut M., Ban N. (2006). Structure of the eukaryotic thiamine pyrophosphate riboswitch with its regulatory ligand. Science.

[49] Abramson J., Adler J., Dunger J., Evans R., Green T., Pritzel A., Ronneberger O., Willmore L., Ballard A.J., Bambrick J. (2024). Accurate structure prediction of biomolecular interactions with AlphaFold 3. Nature.

[50] Nguyen M.N., Sim A., Wan Y., Madhusudhan M.S., Verma C. (2016). Topology independent comparison of RNA 3D structures using the CLICK algorithm. Nucleic Acids Res..

[51] Serganov A., Polonskaia A., Phan A.T., Breaker R.R., Patel D.J. (2006). Structural basis for gene regulation by a thiamine pyrophosphate-sensing riboswitch. Nature.

[52] Wayment-Steele H.K., Kladwang W., Strom A.I., Lee J., Treuille A., Becka A., Das R., Eterna Participants (2022). RNA secondary structure packages evaluated and improved by high-throughput experiments. Nat. Methods.

[53] Adamczyk B., Antczak M., Szachniuk M. (2022). RNAsolo: A repository of cleaned PDB-derived RNA 3D structures. Bioinformatics.

[54] Fu L., Niu B., Zhu Z., Wu S., Li W. (2012). CD-HIT: Accelerated for clustering the next-generation sequencing data. Bioinformatics.

[55] Zhang C., Shine M., Pyle A.M., Zhang Y. (2022). US-align: Universal structure alignments of proteins, nucleic acids, and macromolecular complexes. Nat. Methods.

[56] Schrödinger (2015). The PyMOL Molecular Graphics System, Version 1.8.

[57] Dawson W.K., Maciejczyk M., Jankowska E.J., Bujnicki J.M. (2016). Coarse-grained modeling of RNA 3D structure. Methods.

[58] Leontis N.B., Westhof E. (2001). Geometric nomenclature and classification of RNA base pairs. RNA.

[59] Lu X.J., Bussemaker H.J., Olson W.K. (2015). DSSR: An integrated software tool for dissecting the spatial structure of RNA. Nucleic Acids Res..

[60] Sarver M., Zirbel C.L., Stombaugh J., Mokdad A., Leontis N.B. (2008). FR3D: Finding local and composite recurrent structural motifs in RNA 3D structures. J. Math. Biol..

[61] Ingraham J., Garg V., Barzilay R., Jaakkola T., Wallach H., Larochelle H., Beygelzimer A., d’Alché-Buc F., Fox E., Garnett R. (2019). Generative Models for Graph-Based Protein Design. Advances in Neural Information Processing Systems, Proceedings of the 2019 Conference on Neural Information Processing Systems, Vancouver, BC, USA, 8–14 December 2019.

[62] Williams R.J., Zipser D. (1989). A Learning Algorithm for Continually Running Fully Recurrent Neural Networks. Neural Comput..

[63] Lu X., Olson W.K. (2003). 3DNA: A software package for the analysis, rebuilding and visualization of three-dimensional nucleic acid structures. Nucleic Acids Res..

[64] Fey M., Lenssen J.E. (2019). Fast graph representation learning with PyTorch Geometric. arXiv.

[65] Oliver C., Mallet V., Waldispühl J., Churkin A., Barash D. (2025). 3D-Based RNA Function Prediction Tools in rnaglib. RNA Design: Methods and Protocols.

[66] Bonilla S.L., Kieft J.S. (2022). The promise of cryo-EM to explore RNA structural dynamics. J. Mol. Biol..

